# Sleep in post-COVID-19 recovery period and its impact on different domains of quality of life

**DOI:** 10.1186/s41983-021-00429-7

**Published:** 2021-12-14

**Authors:** Samir El Sayed, Sarah Gomaa, Doaa Shokry, Ahmed Kabil, Ahmed Eissa

**Affiliations:** 1 Department of Psychiatry, Faculty of Medicine, Mansoura University, Mansoura, 35516 Egypt; 2 Mansoura University Students’ Hospital, Mansoura University, Mansoura, Egypt; 3 Department Community Medicine, Faculty of Medicine, Mansoura University, Mansoura, Egypt; 4 Department of Chest Medicine, Faculty of Medicine, Al Azhar University, Cairo, Egypt; 5 Department of Pulmonology Medicine, Hayat National Hospital, Riyadh, Kingdom of Saudi Arabia; 6 Department of Neuropsychiatry, Faculty of Medicine, Port Said University, Port Said, Egypt

**Keywords:** COVID-19, Quality, Recovery, Sleep

## Abstract

**Background:**

COVID-19 pandemic became a global health problem affecting the life of millions of people all over the world. The effects of this pandemic were not only on the physical and medical aspects but also on the psychological issues including anxiety disorders, depressive manifestations, sleep problems and others. Sleep disorders were very commonly reported during the novel Coronavirus-19 pandemic either in the acute phase of COVID-19 infection or after recovery. These sleep problems might have a drastic burden on the recovered patients’ life. This study aimed to investigate the sleep in the post-Coronavirus-19 period and if has an impact on the different items of patients’ quality of life. This cross-sectional observational study investigated the sleep problems in 500 patients in the post recovery period using Insomnia Severity Index and Pittsburgh sleep quality index (PSQI), their relation to this critical period and their impact on different domains of Quality of Life which was assessed by the SF36 Health Survey.

**Results:**

Socio-demographic characteristics of 500 post-Coronavirus-19 patients were collected; the insomnia severity index and Pittsburgh sleep quality index evaluated the sleep pattern. The quality of life was investigated using Short Form 36 scale. The study revealed high scores of insomnia severity index (13.01 ± 4.9), Pittsburgh sleep quality index (15.37 ± 4.43), also high scores of different items of scale of quality of life in the studied group.

**Conclusion:**

Post-COVID-19 sleep disturbances were commonly reported in the recovery period, also these sleep deficits had an impact on the physical and mental aspects of quality of life, so these sleep problems must be managed properly especially in this critical pandemic era.

## Background

Prior to 2020, the deaths caused by respiratory infection was on the 4th leading causes [1] but after the start of COVID-19 pandemic more death cases related to this global health problem [2]. According to the World Health Organization (WHO), the COVID-19 infection became a worldwide devastating health issue starting in December 2019 in China and then gradually was a global pandemic [3].

Due to absence of specific treatment, the world health systems began to be affected, now the vaccines are available for COVID-19 but at the same time their efficacy still of doubt [4, 5].

COVID-19 has double weapon on physical and mental domains of health including grief from loss, financial issues, social curfew and ambiguity about the future [6, 7].

Generally speaking, mental health problems, including depressive symptoms and anxiety disorders, have had negatively affected the general population during this pandemic [8, 9].

Studies showed that mental health problems, such as depression, anxiety, insomnia, and post-traumatic stress disorder (PTSD), dramatically increased after the COVID-19 pandemic: 53.8% of respondents had the psychological impact as moderate or severe; 16.5% of participants reported moderate to severe depressive manifestations; 28.8% of participants had moderate to severe anxiety symptoms; and 24.5% of participants showed psychological distress [10].

The WHO declared that quarantine decreased the total number of COVID-19 positive cases but also leading to emergence of fearful reaction, stressful condition, significant anxiety and sleep disorders among general population [11].

The quality of life (QoL) has been explored previously in studies investigating the noncommunicable and chronic diseases. It was described as “a patient’s general subjective feeling of the burden of illness or medical condition on different aspects including physical, psychological, social, and occupational functioning [12].

Researches suggest that QoL is a significant factor of persistence in general health and well-being [13].

Pandemics of infectious diseases, such as COVID-19, negatively affect the physical, social, and psychological capacities of people and societies, and have significant economic impacts [14, 15].

A study from Morocco concluded negative implications of the COVID-19 pandemic on Health Related Quality of Life [16].

Though recent studies have cautioned about the psychological consequences of massive lockdown to control COVID-19 spread on individuals’ QoL, the researches studying the effects of the COVID-19 pandemic on various domains of QoL in different countries are not enough [17].

Recently, the association between physical disease and mental health has taken a crucial role, because the detrimental psychiatric condition might have a considerable impact on the individual’s quality of life [18, 19].

Sleep is an important biological mechanism for maintaining internal homeostasis and quality of life. Increased sleep quality has positive results on physical and mental health [20], sleep problems negatively affected the immune responses by their effects on the circadian rhythm of the body [21].

A study concluded that dysregulation of circadian rhythm and sleep may be associated with higher risk of SARS-CoV-2 infection and the severity of its clinical presentation [22].

Sleep disorders have been associated to infectious disease hazard, the incidence and progression of many diseases including depressive disorder [23].

Although there were studies showing impaired sleep quality in hospitalized COVID-19 cases but investigating sleep problems in recovered patients needed more studies [24].

Sleep disturbance may be associated with the adverse health effects of COVID-19 patients. Compared to those without sleep disturbance, COVID-19 patients who suffered from sleep disturbance had a higher incidence of hospital-acquired infection, longer hospitalization days, and an increased need for admission in ICU care than those without sleep disturbance [25].

PTSD after recovery from COVID-19 has been correlated to sleep problems, high anxiety level and depressive manifestations in Chinese and Italian people, also the quality of life of front liners workers and patients was extremely burdened during the post recovery period [26].

Several studies have shown that proper sleep not only attenuate the hazardous effect of non-communicable diseases (NCDs) [27], but also leading to enhanced immunity to protect against different viral infections, so with appropriate sleep structure, the enhanced immune system reduced the possibility of COVID-19 infection [28].

The present study aimed to study the sleep problems in post-COVID-19 patients and their impact on different domains of quality of life.

## Methods

Study design which included a single Centre cross-sectional observational study, from the 1st of August 2020 till 30th of November 2020 for investigating the sleep problems in patients in the post-COVID-19 recovery period, their relation to this critical period and their impact on different domains of Quality of Life (QOL).

Study population which was composed of a sample of 500 patients of COVID-19 after 2 consecutive negative Polymerase Chain Reaction (PCR) tests within 1 months after recovery who presented for pulmonology clinic for reevaluation after recovery and psychiatric outpatient clinic for evaluation regarding their complaints about sleep problems were randomly selected to participate in this study.

The studied group of patients have the criteria of being both sexes, age ranged from 18 to 60 years and must have two negative PCR tests for COVID 19.

Patients with well-known psychiatric disorders and under the effect of psychotropic medications were excluded from the study.

Socio-demographic and clinical data form which was used in the study based on clinical experience and the knowledge derived from the scientific sources aiming to study the objectives of this study. The semi-structured form included socio-demographic data, such as age, gender, marital status, education level, occupation status, residence and clinical data.

Insomnia Severity Index (ISI) which is a brief scale evaluating the patient's insomnia. The ISI evaluates the subjective complaints and results of insomnia as well as the level of dysfunctions from these sleep disturbances. The ISI is composed of seven domains which include the following: (a) the degree of severity of sleep-onset (initial), (b) The maintenance of sleep (middle), (c) early morning awakening (terminal) problems, (d) to what extent the patient was satisfied with current sleep pattern, (e) impact on daily activities, (f) observed by others/interfering with the quality of life and (g) distress level caused by the sleep problem. Each item is scaled on a 5-point Likert scale from 0 to 4, so the total score ranging from 0 to 28.

Interpretation of the results is as follows: absence of insomnia (0–7); subthreshold insomnia (8–14); moderate insomnia (15–21); and severe insomnia (22–28) [29].

Pittsburgh sleep quality index (PSQI) which is a scale that study the subjective sleep quality and different domains of sleep over a period of 1-month interval through 19 items. The subdomains of the index include subjective sleep quality, latency of sleep, sleep duration, habitual sleep efficiency, sleep disturbance, use of sleeping medicines, and daytime impacts. The results of the index were estimated on a scoring scale from 0 to 3, all the sub domains were summated to form the total index score. The total score is from 0 to 21 and, scores equal or greater than 5 indicate a disturbed quality of sleep. PSQI is a valid and reliable international scale to assess subjective quality of sleep [30].

Quality Of Life (QOL) by the SF36 Health Survey is a 36-item-report survey that evaluate eight domains of physical and mental wellbeing ranging from 0 to 100, where the highest score indicates the optimal heath related quality of life (HRQoL) and the lowest score indicates the poor level of HRQoL. The eight domains are physical functioning, role limitations because of physical health problems (role-physical), bodily pain, general health perceptions, vitality, social functioning, role limitations because of emotional problems (role-emotional) and general mental health [31]. The physical health composed of the first four domains and other four domains constitute the mental wellbeing [32].

Statistical analysis which was composed of the Statistical Package of Social Science (SPSS) program for Windows (Standard version 24, IBM Corp. Released 2016. IBM SPSS Statistics for Windows, Version 24.0. Armonk, NY: IBM Corp.) analyzed the Data collected. One-sample Kolmogorov–Smirnov test tested first the normality of data and qualitative data were explained by number and percent. ANOVA test was used to compare more than 2 means, while Kruskal–Wallis test was used to compare more than 2 medians. Continuous variables were illustrated as follows:—Mean ± SD (standard deviation) for parametric data and Median (min–max) for non-parametric data, at the same time, *t* test (parametric) and Mann Whitney test (non-parametric) compared between the two groups. Pearson correlation (parametric) and Spearman correlation (non-parametric) were used to correlate continuous data and finally the results as considered statistically significant when (*p* ≤ 0.05).

## Results

Sociodemographic characteristic of the studied group: included Table 1 which showed socio-demographic characteristics of the patients in which the age was 36.98: SD ± 10.87, the sample of the study composed of 305 males: 61.0% and 195 females: 39.0%, 342 of the studied group were married: 68.4% and 158:31.6% were single.

**Table 1:** Socio-demographic data among the studied group

Socio-demographic data	The studied group (*n* = 500)
Age (years)	
Mean ± SD	36.98 ± 10.87
Range	19–59
Gender	
Male	305 (61.0%)
Female	195 (39.0%)
Marital status	
Married	342 (68.4%)
Single	158 (31.6%)
Education	
Primary	101 (20.2%)
Secondary	213 (42.6%)
University	186 (37.2%)
Occupation	
Worker	302 (60.4%)
Non worker	198 (39.6%)
Residence	
Urban	303 (60.6%)
Rural	197 (39.4%)
Smoking	
Smokers	299 (59.8%)
Non smokers	201 (40.2%)
Duration after 2 consecutive Polymerase Chain Reaction negative swab tests Mean ± SD	22.82 ± 2.79

*SD* standard deviation

Regarding education in which 101:20.2% were primary, 213:42.6% were secondary and 186:37.2% were university. In regard to employment: 302:60.4% were employed and 198:39.6% were unemployed. 303 60.6% were from urban areas and 197:39.4% were from rural areas.

299:59.8% were smokers and 201:40.2% were nonsmokers, the mean duration after 2 consecutive negative PCR swabs was 22.82: SD ± 2.79.

**Fig. 1:**
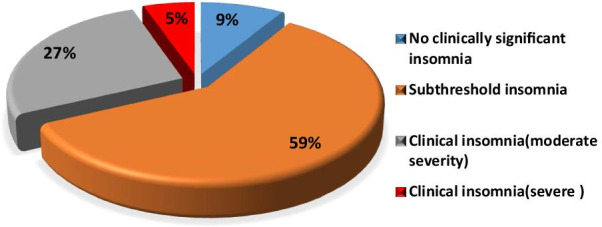
Distribution of the patients according to insomnia severity index

Assessment of sleep by insomnia severity index and Pittsburgh sleep quality index included Table 2 which illustrated the mean score of insomnia severity index was 13.01 ± 4.9, No clinically significant insomnia was 44:8.8%, subthreshold insomnia was 296:59.2%, clinical insomnia (moderate severity) was 133:26.6% and clinical insomnia (severe) was 27:5.4% (Fig. 1).

**Table 2:** Insomnia severity index and components of Pittsburgh sleep quality index

	The studied group (*n* = 500)
Score of insomnia severity index	13.01 ± 4.9
0–7 = No clinically significant insomnia	44 (8.8%)
8–14 = subthreshold insomnia	296 (59.2%)
15–21 = clinical insomnia (moderate severity)	133 (26.6%)
22–28 = clinical insomnia (severe)	27 (5.4%)
Global PSQI Score	15.37 ± 4.43
Global PSQI Score components	
Subjective sleep quality	2.10 ± 0.94
Sleep latency	2.35 ± 0.74
Sleep duration	2.16 ± 0.85
Sleep efficiency	2.15 ± 0.86
Sleep disturbance	2.18 ± 0.85
Use of sleep medication	2.23 ± 0.83
Daytime dysfunction	2.21 ± 0.79

*PSQI* Pittsburgh sleep quality index

Regarding Pittsburgh sleep quality index, Component 1: subjective sleep quality was 2.10 ± 0.94, Component 2: Sleep latency was 2.35 ± 0.74, Component 3: Sleep duration was 2.16 ± 0.85, Component 4: Sleep efficiency was 2.15 ± 0.86, Component 5: Sleep disturbance was 2.18 ± 0.85, Component 6: Use of sleep medication was 2.23 ± 0.83, Component 7: daytime dysfunction was 2.21 ± 0.79 and Global Pittsburgh Sleep Quality Index Score: Sum of seven component scores was 15.37 ± 4.43.

Results of Quality Of Life SF36 in Table 3 which showed components of quality of life SF36 in which Physical functioning was 60:0–100, role limitation due to physical health was 50:0–100, role limitation due to emotional problems was 33.33 (0–100), energy/fatigue was 10:0–100, emotional well-being was 8:0–100, social functioning was 12.5:0–100, pain was 0:0–100 and general health was 15:0–100.

**Table 3:** Different domains of quality of life SF 36 scale

SF36	The studied group (*n* = 500)
Physical functioning	60 (0–100)
Role limitation due to physical health	50 (0–100)
Role limitation due to emotional problems	33.33 (0–100)
Energy/ fatigue	10 (0–100)
Emotional well being	8 (0–100)
Social functioning	12.5 (0–100)
Pain	0 (0–100)
General health	15 (0–100)

Data were expressed as median (min–max)

*SF 36* Quality of life Short Form 36

**Fig. 2:**
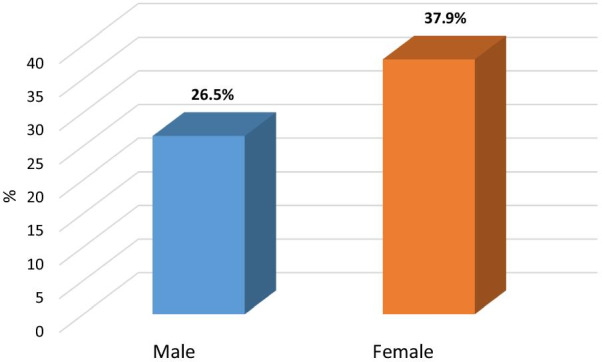
Gender of the patients as predictor of moderate and severe insomnia

**Fig. 3:**
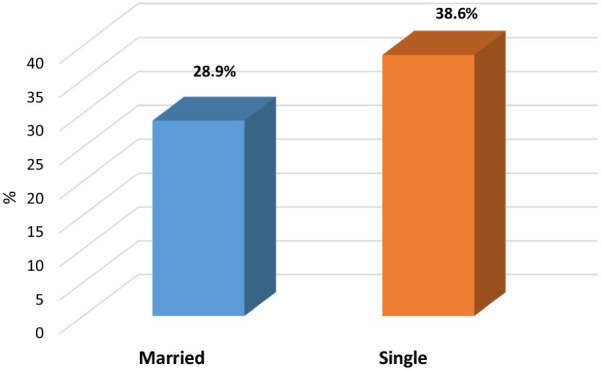
Marital status of the patients as predictor for moderate and severe insomnia

**Fig. 4:**
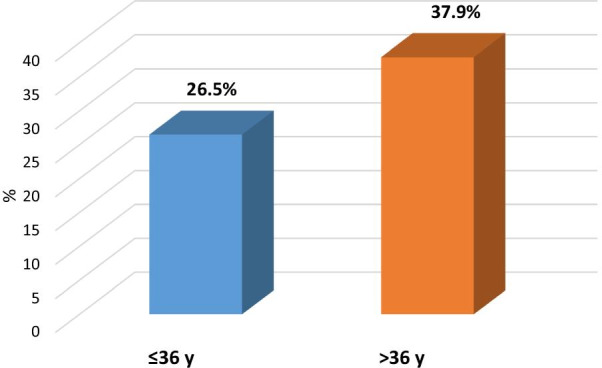
Age of the patients as predictor of moderate and severe insomnia

Association between moderate, severe insomnia and patients’ characteristics elaborated in Table 4 presented association between socio-demographic data and moderate, severe insomnia in which there was a statistically significant positive correlation between age of the patients and moderate severe insomnia, *p* value = 0.006*, OR (95%CI) = 1.7:1.2–2.5, also there was a statistically significant positive correlation between female gender of patients and moderate severe insomnia, *p* value = 0.013*, OR (95%CI) = 1.6:1.1–2.4, single of marital status, *p* value = 0.031*, OR (95%CI) = 1.5:1.04–2.3, days after recovery from COVID 19, *p* value ≤ 0.001*, Physical functioning, *p* value: 0.025*, OR (95%CI): 1.5 (1.1–2.3.), Role limitation due to physical health, *p* value: 0.009*, OR (95%CI): 1.7 (1.1–2.5), Role limitation due to emotional problems, *p* value ≤ 0.001*, OR (95%CI): 2.5 (1.6–3.8), General health, *p* value: ≤ 0.001*, OR (95%CI): 2.1 (1.4–3.1), Global PSQI Score, *p* value: ≤ 0.001*, OR (95%CI): 57.6 (23–144) (Figs. 2, 3, 4).

**Table 4:** Association between moderate, severe insomnia and patients’ characteristics

Socio-demographic data	Total (*n* = 500)	Moderate and severe insomnia (*n* = 160)	*χ*^2^ (*p* value)	OR (95%CI)
Age/ years				
Mean ± SD	36.98 ± 10.87			
≤ 36 y (r)	260 (52%)	69 (26.5%)	7.42 (0.006*)	1 1.7 (1.2–2.5)
> 36 y	240 (48%)	91 (37.9%)		
Gender				
Male (r)	305 (61.0%)	97 (27.9%)	6.1 (0.013*)	1 1.6 (1.1–2.4)
Female	195 (39.0%)	63 (38.5%)		
Marital status				
Married (r)	342 (68.4%)	99 (28.9%)	4.63 (0.031*)	1 1.5 (1.04–2.3)
Single	158 (31.6%)	61 (38.6%)		
Education				
Primary (r)	101 (20.2%)	30 (29.7%)	–	1
Secondary	213 (42.6%)	70 (32.9%)		1.2 (0.7–1.9)
University	186 (37.2%)	60 (32.3%)	0.32 (0.85)	1.1 (0.6–1.9)
Occupation				
Worker	302 (60.4%)	103 (34.1%)	1.55 (0.21)	1.28 (0.9–1.8)
Non worker (r)	198 (39.6%)	57 (28.8%)		1
Residence				
Urban (r)	303 (60.6%)	90 (29.7%)	1.86 (0.17)	1
Rural	197 (39.4%)	70 (35.5%)		1.3 (0.8–1.9)
Smoking				
Smokers (r)	299 (59.8%)	88 (29.4%)	2.26 (0.13)	1
Non smokers	201 (40.2%)	72 (35.8%)		1.3 (0.9–2)
Days after recovery from COVID 19	13.04 ± 4.99			
≤ 12 days (r)	284 (56.8%)	0 (0%)	309 (≤ 0.001*)	NA
> 12 days	216 (43.2%)	160 (74.1%)		
Physical functioning	60 (0–100)			
≤ 60	254 (50.8%)	93 (36.6%)	5.05 (0.025*)	1.5 (1.1–2.3.)
> 60 (r)	246 (49.2%)	67 (27.2%)		1
Role limitation due to physical health	50 (0–100)			
≤ 50	312 (62.4%)	113 (36.2%)	6.78 (0.009*)	1.7 (1.1–2.5)
> 50 (r)	188 (37.6%)	47 (25.0%)		1
Role limitation due to emotional problems	33.33 (0–100)			
≤ 33.33	322 (64.4%)	124 (38.5%)	17.61 (≤ 0.001*)	2.5 (1.6–3.8)
> 33.33 (r)	178 (35.6%)	36 (20.2%)		1
Energy/ fatigue	10 (0–70)			
≤ 10	308 (61.6%)	93 (30.2%)	1.20 (0.27)	0.23 (0.8–1.8)
> 10 (r)	192 (38.4%)	67 (34.9%)		1
Emotional well being	8 (0–56)			
≤ 8	293 (58.6%)	94 (32.1%)	0.002 (0.96)	1.01 (0.6–1.5)
> 8 (r)	207 (41.4%)	66 (31.9%)		1
Social functioning	12.5 (0–100)			
≤ 12.5	268 (53.6%)	78 (29.1%)	2.23 (0.14)	1.3 (0.9–1.9)
> 12.5 (r)	232 (46.4%)	82 (35.3%)		1
Pain	0 (0–100)			
0	288 (57.6%)	85 (29.5%)	1.93 (0.17)	1.3 (0.9–1.9)
> 0 (r)	212 (42.4%)	75 (35.4%)		1
General health	15 (0–90)			
≤ 15	285 (57.0%)	110 (38.6%)	13.25 (≤ 0.001*)	2.1 (1.4–3.1)
> 15 (r)	215 (43.0%)	50 (23.3%)		1
Global PSQI Score	15.37 ± 4.43			
≤ 17	275 (55.0%)	155 (56.6%)	168.2 (≤ 0.001*)	57.6 (23–144)
> 17 (r)	225 (45.0%)	5 (2.2%)		1

Continuous variables were divided according to median value

*Statistically significant results

(r): reference group, *OR* odds ratio, *CI* confidence interval, *PSQI* Pittsburgh sleep quality index, *SD* standard deviation

Table 5 illustrates the correlation among insomnia severity index score, global Pittsburgh quality sleep score and other variables including age, mean duration after 2 consecutive negative swab and quality of life scale SF 36 in which:

**Table 5:** Correlation between score of insomnia severity index, global PSQI Score and other variables

	Score of insomnia severity index		Global PSQI Score	
	*r*	*p*	*r*	*p*
Age	0.028	0.537	− 0.031	0.482
Days after recovery from COVID 19	0.985	≤ 0.001*	0.897	≤ 0.001*
Physical functioning	− 0.840	≤ 0.001*	− 0.773	≤ 0.001*
Role limitation due to physical health	− 0.198	≤ 0.001*	− 0.186	≤ 0.001*
Role limitation due to emotional problems	− 0.164	≤ 0.001*	− 0.130	0.004*
Energy/ fatigue	0.010	0.828	0.013	0.772
Emotional well being	0.005	0.909	0.019	0.672
Social functioning	0.042	0.348	− 0.056	0.215
Pain	0.045	0.320	− 0.033	0.457
General health	− 0.397	≤ 0.001*	− 0.384	≤ 0.001*
Global PSQI Score	− 0.888	≤ 0.001*	–	–

*Statistically significant results

*R* reference group, *F* ANOVA test, *t* student *t* test, *PSQI* Pittsburgh sleep quality index

There was a statistically significant negative correlation between mean duration after recovery from COVID-19, insomnia severity index scale, *p* value ≤ 0.001*.

Also, there was a statistically significant positive correlation between insomnia severity index scale and different domains of quality of life scale SF 36 including physical functioning, *p* value ≤ 0.001*, role limitation due to physical health, *p* value ≤ 0.001*, role limitation due to emotional problems and general health, *p* value ≤ 0.001*.

There was a statistically significant positive correlation between insomnia severity index scale and global Pittsburgh quality sleep score, *p* value ≤ 0.001*.

Also there was a statistically significant positive correlation between global Pittsburgh quality sleep score and mean duration after recovery from COVID-19, *p* value ≤ 0.001*, different domains of quality of life scale SF 36 including physical functioning value ≤ 0.001*, role limitation due to physical health, *p* value ≤ 0.001*, role limitation due to emotional problems and general health, *p* value ≤ 0.001*.

Multivariate regression analysis for independent predictors of Moderate and severe insomnia in the studied group included in Table 6 which illustrated the Multivariate regression analysis for independent predictors of Moderate and severe insomnia in the studied group in which age of the patient has *p* value 0.022, OR (95% CI) 1.9 (1.1–3.3), gender of the patient in which female patients has *p* value 0.049, OR (95% CI) 1.7 (1.01–2.9), marital status in single patients has *p* value 0.012, OR (95% CI) 2.2 (1.2–3.9), physical functioning ≤ 60, *p* value 0.017, OR (95% CI) 1.9 (1.12–3.3), role limitation due to physical health ≤ 50 has *p* value 0.002, OR (95%CI) 2.3 (1.4–4.1), role limitation due to emotional problems ≤ 33.33 has *p* value ≤ 001, OR (95%CI) 3.0 (1.7–5.4), general health ≤ 15 has *p* value 0.042, OR (95% CI) 1.8 (1.02–3.1) and global PSQI Score ≤ 17 has *p* value ≤ 0.001, OR (95% CI) 79.6 (30–210).

**Table 6:** Multivariate regression analysis for independent predictors of Moderate and severe insomnia

Independent predictors	Multivariate regression analysis			
	*β*	SE	*p* value	OR (95% CI)
Age/ years > 36 y	0.649	0.284	0.022	1.9 (1.1–3.3
Gender Female	0.539	0.274	0.049	1.7 (1.01–2.9)
Marital status Single	0.766	0.306	0.012	2.2 (1.2–3.9)
Physical functioning ≤ 60	0.649	0.272	0.017	1.9 (1.12–3.3)
Role limitation due to physical health ≤ 50	0.854	0.279	0.002	2.3 (1.4–4.1)
Role limitation due to emotional problems ≤ 33.33	1.112	0.290	≤ 0.001	3.0 (1.7–5.4)
General health ≤ 15	0.568	0.280	0.042	1.8 (1.02–3.1)
Global PSQI Score ≤ 17	4.377	0.496	≤ 0.001	79.6 (30–210)

Continues variables were divided according to median value

*SE* standard error, *OR* odds ratio, *CI* confidence interval, *PSQI* Pittsburgh sleep quality index

## Discussion

The current study investigated the sleep problems in post-COVID-19 patients in the recovery period and their impact on different subdomains of quality of life. To our knowledge this study is one of leading studies examining the sleep problems in the recovery period after COVID-19 infection and their implication on quality of life.

The current study revealed high mean score of insomnia severity index which was in agreement with the study noting COVID-19 patients after recovery will still have increasing levels of depressive manifestation, anxiety level, stressful condition, decreased sleep quality and impaired QoL [33].

On the contrary [26] reported a much lower prevalence of poor sleep quality 18.2% in 7236 self-selected Chinese volunteers. They assessed the sleep quality using the PSQI; however, they used a higher cutoff point) > 7, leading to underestimation of the sleep problems in the participated population.

The concurrent study found high score of global Pittsburgh quality sleep scale in the post-COVID-19 patients which is in accordance with the study concluding the persistent manifestations after COVID-19 infection were anxiety, depressive disorder and sleep quality insufficiency assessed by Pittsburgh quality sleep score, but this study was conducted in the medical staff only and not generalized in the general population [34].

In a case series, three out of four patients had worsening in subjective sleep quality and sleep problems, including changes in subjective sleep quality, sleep latency and daytime function, were observed in (85%) of patients who recovered from COVID-19 infection and were evaluated again 8 weeks after discharge[35].

This study concluded that the post-COVID-19 patients showed impairment of different subdomains of quality of life including physical and mental aspects which in agreement with the study highlighting outcomes in ICU COVID‐19 patients including deleterious effects on physical health and quality of life [36].

The current study found a statistically significant positive association between patients from urban areas and mean insomnia severity index which in accordance with the research highlighted post-COVID-19 cases developing sleep problems were more frequent in urban areas and cities in relation to rural places [26].

This study revealed a statistically significant negative association between mean duration after recovery from COVID-19, insomnia severity index scale and which in line with the study mentioning sleep quality level and insomnia were more evident in the time period shortly after recovery from COVID-19 [26].

Also this finding was in accordance with results of a study noted that after critical illness, especially after intensive care, sleep disturbance is quite common even up to 1 year [37].

This study found a statistically significant positive association between insomnia severity index scale and different domains of quality of life scale SF 36 including physical health, role restriction because of physical health, role limitation due to emotional burdens and general wellbeing which in parallel with the results of the study concluded impaired quality of life (QoL) independently related to high anxiety score, severe depressive manifestations, poorer quality of sleep and insomnia problem [38].

Patients in whom the COVID-19 onset was > 12 weeks ago, there was still a major persisting impact on QoL across all domains in both survivors and family members and this evidenced the severe impact of post-acute COVID-19 (‘long COVID-19’) and ‘chronic COVID-19 [39].

Also the study has predictors of moderate and severe insomnia in the studied group including age and female gender which in agreement with studies recent studies from China and Italy which revealed that females are more vulnerable to sleep problems compared to males, and that younger age groups had a higher tendency to have more impairment of QOL [18, 40].

The most evidence-based treatment is cognitive behaviour therapy (CBT), especially Internet CBT that can prevent the spread of infection during the pandemic.

Also use of Cognitive Behavior Therapy (CBT) to treat psychiatric symptoms during COVID-19 by helping the patients to combat anxiety with the use of relaxation techniques and prevent depression onset by altering the schedule of their routine activities [41].

Internet Cognitive Behavioral Therapy (CBT) can treat insomnia:

There was a strong support for the effectiveness of digital CBT-I in treating insomnia. dCBT-I has potential to elaborate the use of CBT-I, improving the accessibility and availability of CBT-I content for insomnia patients worldwide particularly in the era of COVID-19 pandemic [42].

In conclusion, the sleep problems were common among patients in the post recovery period after COVID-19, also these sleep problems affected the different domains of quality of life of those patients. In addition, the question evolved if these sleep manifestations are transient or persist for a long period, this needs more research.

## Conclusions

In conclusion the study revealed high score of insomnia and sleep disturbances during the recovery period of COVID-19 infection, these sleep problems have drastic implications of different domains of quality of life in which they must be managed during this critical era of COVID-19.

### Study limitations

This is a single-centre cross-sectional study so we need follow-up studies and multicenter studies to get more consolidated and generalized data, also this study was descriptive so we need for interventional studies to find the new plan of management and treatment for these common sleep problems.

## Data Availability

The data sets used and/or analysed during the current study are available from the corresponding author on reasonable request.

## Abbreviations

WHO: World Health Organization
COVID-19: Coronavirus diseases-19
PTSD: Post-traumatic stress disorder
NCDs: Non communicable diseases
QOL: Quality of life
PCR: Polymerase chain reaction
ISI: Insomnia severity index
PSQI: Pittusberg sleep quality index
DCBT: Digital cognitive behavioral therapy

## References

[1] Nowbar AN, Gitto M, Howard JP, Francis DP, Al-Lamee R (2019). Mortality from ischemic heart disease. Circ Cardiovasc Qual Outcomes.

[2] WHO. Coronavirus diseases 2019 (COVID-19): situation report, 72, Switzerland: World Health Organization; 2020.

[3] Cucinotta D, Vanelli M (2020). WHO declares COVID-19 a pandemic. Acta Biomed.

[4] Hotez PJ, Corry BD, Bottazzi ME (2020). COVID-19 vaccine design: the Janus face of immune enhancement. Nat Rev Immun.

[5] Lurie N, Sharfstein JM, Goodman JL (2020). The development of COVID-19 vaccines: safeguards needed. JAMA.

[6] Berg-Weger M, Morley JE (2020). Editorial: loneliness and social isolation in older adults during the COVID-19 pandemic: implications for gerontological social work. J Nutr Health Aging.

[7] Dubey S, Biswas P, Ghosh R, Chatterjee S, Dubey MJ, Chatterjee S, Lahiri D, Lavie CJ (2020). Psychosocial impact of COVID-19. Diabetes Metab Syndr.

[8] Choi EPH, Hui BPH, Wan EYF (2020). Depression and anxiety in Hong Kong during COVID-19. Int J Environ Res Public Health.

[9] Yang Y, Li W, Zhang Q, Zhang L, Cheung T, Xiang YT (2020). Mental health services for older adults in China during the COVID-19 outbreak. Lancet Psychiatry.

[10] Jinglong Z, Rong S, Juan Y (2020). Anxiety and depression in elderly patients during epidemic of coronavirus disease 2019 and its influencing factors. J Clin Med Pract.

[11] Wang C, Chudzicka-Czupała A, Tee ML (2021). A chain mediation model on COVID-19 symptoms and mental health outcomes in Americans, Asians and Europeans. Sci Rep.

[12] Haraldstad K, Network TL, Wahl AK, Andenæs R, Andersen JR, Andersen MH, Beisland E, Borge CR, Engebretsen E, Eisemann M (2019). A systematic review of quality of life research in medicine and health sciences. Qual Life Res.

[13] Fayers PM, Machin D (2015). Quality of life: the assessment, analysis, and interpretation of patient-reported outcomes.

[14] Qiu J, Shen B, Zhao M, Wang Z, Xie B, Xu Y (2020). A nationwide survey of psychological distress among Chinese people in the COVID-19 epidemic: implications and policy recommendations. Gen Psychiatr.

[15] Yezli S, Khan A (2020). COVID-19 social distancing in the Kingdom of Saudi Arabia: Bold measures in the face of political, economic, social and religious challenges. Travel Med Infect Dis.

[16] Mucci F, Mucci N, Diolaiuti F (2020). Lockdown and isolation: psychological aspects of COVID-19 pandemic in the general population. Clin Neuropsychiatry.

[17] Rubin GJ, Wessely S (2020). The psychological effects of quarantining a city. BMJ.

[18] Wang C, Pan R, Wan X, Tan Y, Xu L, Ho CS, Ho RC (2020). Immediate psychological responses and associated factors during the initial stage of the 2019 Coronavirus Disease (COVID-19) epidemic among the general population in China. Int J Environ Res Public Health.

[19] Biddle SJ, Ciaccioni S, Thomas G, Vergeer I (2019). Physical activity and mental health in children and adolescents: an updated review of reviews and an analysis of causality. Psychol Sport Exerc.

[20] Vitale JA, Perazzo P, Silingardi M, Biffi M, Banfi G, Negrini F (2020). Is disruption of sleep quality a consequence of severe COVID-19 infection? A case-series examination. Chronobiol Int.

[21] Ono BHVS, Souza JC (2020). Sleep and immunity in times of COVID-19. Rev Assoc Med Bras.

[22] Meira ECM, Miyazawa M, Gozal D (2020). Putative contributions of circadian clock and sleep in the context of SARS-CoV-2 infection. Eur Respir J.

[23] Irwin MR (2015). Why sleep is important for health: a psychoneuroimmunology perspective. Annu Rev Psychol.

[24] Liu K, Chen Y, Wu D, Lin R, Wang Z, Pan L (2020). Effects of progressive muscle relaxation on anxiety and sleep quality in patients with COVID-19. Complement Ther Clin Pract.

[25] Zhang J, Xu D, Xie B, Zhang Y, Huang H, Liu H, Chen H, Sun Y, Shang Y, Hashimoto K, Yuan S (2020). Poor-sleep is associated with slow recovery from lymphopenia and an increased need for ICU care in hospitalized patients with COVID-19: a retrospective cohort study. Brain Behav Immun.

[26] Huang Y, Zhao N (2020). Generalized anxiety disorder, depressive symptoms and sleep quality duringCOVID-19 outbreak in China: a web-based cross-sectional survey. Psychiatry Res.

[27] Lucassen EA, Rother KI, Cizza G (2012). Interacting epidemics? Sleep curtailment, insulin resistance, and obesity. Ann N Y Acad Sci.

[28] Paules CI, Marston HD, Fauci AS (2020). Coronavirus infections—more than just the common cold. JAMA.

[29] Bastien CH, Vlières A, Morin CM (2001). Validation of the Insomnia Severity Index as an outcome measure for insomnia research. Sleep Med.

[30] Buysse DJ, Reynolds CF, Monk TH, Berman SR, Kupfer DJ (1989). The Pittsburgh Sleep Quality Index: a new instrument for psychiatric practice and research. Psychiatry Res.

[31] Ware JE, Sherbourne CD (1992). The MOS 36-item short-form health survey (SF-36). I. Conceptual framework and item selection. Med Care.

[32] Milic M, Gazibara T, Pekmezovic T, Kisic Tepavcevic D, Maric G, Popovic A, Stevanovic J, Patil KH, Levine H (2020). Tobacco smoking and health-related quality of life among university students: mediating effect of depression. PLoS ONE.

[33] Sood S (2020). Psychological effects of the Coronavirus disease-2019 pandemic. RHiME.

[34] Lai J, Ma S, Wang Y (2020). Factors associated with mental health outcomes among health care workers exposed to coronavirus disease 2019. JAMA Netw Open.

[35] Mahmoudi H, Saffari M, Movahedi M, Sanaeinasab H, Rashidi-Jahan H, Pourgholami M, Poorebrahim A, Barshan J, Ghiami M, Khoshmanesh S, Potenza MN, Lin CY, Pakpour AH (2021). A mediating role for mental health in associations between COVID-19-related self-stigma, PTSD, quality of life, and insomnia among patients recovered from COVID-19. Brain Behav.

[36] Stam H, Stucki G, Bickenbach J (2020). COVID-19 and post intensive care syndrome: a call for action. J Rehabil Med.

[37] Lutchmansingh DD, Knauert MP, Antin-Ozerkis DE, Chupp G, Cohn L, Dela Cruz CS, Ferrante LE, Herzog EL, Koff J, Rochester CL, Ryu C, Singh I, Tickoo M, Winks V, Gulati M, Possick JD (2021). A clinic blueprint for post-coronavirus disease 2019 RECOVERY: learning from the past looking to the future. Chest.

[38] Greenberg N, Docherty M, Gnanapragasam S, Wessely S (2020). Managing mental health challenges faced by healthcare workers during COVID-19 pandemic. BMJ.

[39] Greenhalgh T, Knight M, Court C, Buxton M, Husain L (2020). Management of post-acute covid-19 in primary care. BMJ.

[40] Mazza C, Ricci E, Biondi S, Colasanti M, Ferracuti S, Napoli C (2020). Nationwide survey of psychological distress among Italian people during the COVID-19 pandemic: Immediate psychological responses and associated factors. Int J Environ Res Public Health.

[41] Ho CS, Chee CY, Ho RC (2020). Mental health strategies to combat the psychological impact of coronavirus disease 2019 (COVID-19) beyond paranoia and panic. Ann Acad Med Singap.

[42] Soh HL, Ho RC, Ho CS, Tam WW (2020). Efficacy of digital cognitive behavioural therapy for insomnia: a meta-analysis of randomized controlled trials. Sleep Med.

